# Supplementary material for: Genome skimming reveals novel plastid markers for the molecular identification of illegally logged African timber species
Source: PLoS One. 2021 Jun 11;16(6):e0251655. doi: 10.1371/journal.pone.0251655 (PMC8195358; doi:10.1371/journal.pone.0251655)
Supplement: S2 Table — (DOCX) [file pone.0251655.s002.docx]

| **Family** | **NCBI Reference Sequence** | **Description** |
| --- | --- | --- |
| Ebenaceae | NC_033502.1 | Diospyros blancoi voucher PDBK 2014-0240 chloroplast, complete genome |
| Sapotaceae | NC_033501.1 | Pouteria campechiana voucher PDBK 2014-0238 chloroplast, complete genome |
| Sapotaceae | NC_041130.1 | Sideroxylon wightianum chloroplast, complete genome |
| Moraceae | NC_047236.1 | Morus celtidifolia chloroplast, complete genome |
| Meliaceae | NC_037362.1 | Khaya senegalensis isolate KS chloroplast, complete genome |
| Meliaceae | NC_037250.1 | Entandrophragma cylindricum isolate c-5-ENTC-46 chloroplast, complete genome |
| Fabaceae | NC_016708.2 | Millettia pinnata chloroplast, complete genome |
| Fabaceae | NC_036742.1 | Guibourtia leonensis voucher P:HB6561 chloroplast, complete genome |
| Fabaceae | NC_047329.1 | Afzelia quanzensis voucher Yi14511-KUN plastid, complete genome |
| Fabaceae | NC_045106.1 | Ormosia semicastrata isolate BOP217157 chloroplast, complete genome |
| Fabaceae | NC_049084.1 | Pterocarpus santalinus isolate TXZT chloroplast, complete genome |
