# Supplementary material for: Genome skimming reveals novel plastid markers for the molecular identification of illegally logged African timber species
Source: PLoS One. 2021 Jun 11;16(6):e0251655. doi: 10.1371/journal.pone.0251655 (PMC8195358; doi:10.1371/journal.pone.0251655)
Supplement: Attachment — Submitted filename: Response to Reviewers.pdf [file pone.0251655.s021.pdf]

MAURIZIO MASCARELLO
Meise Botanic Garden
Nieuwelaan 38, 1860 Meise, Belgium
maurizio.mascarello.88@gmail.com

Prof. Tzen-Yuh Chiang
Academic editor
PLOS ONE

March 24th, 2021

Object: Response to reviewers

Dear Prof. Tzen-Yuh Chiang,

Thanks a lot for the feedback given in your email of 04/03/2021. After careful evaluation and discussion with co-authors, I would like to provide you with an answer to various comments made by the reviewer:

1) In section "5. Reviewer Comments to Authors", reviewer1 mentioned that "*Authors overemphasized the polymorphisms for these timber species, and only compared the variations between related species".* We do not agree with this comment as we firstly performed multiple alignments at the family level to evaluate the genetic variation in the conventional plastid markers mentioned in the manuscript. Once we found low polymorphic variation in the *matK* and/or in the *rbcL* regions (a maximum of 5 SNPs and 3 SNPs, respectively), we selected closely-related species for the search for highly-variable genetic regions.

2) Next, reviewer1 stated that "*For the application of forensic species identification, authors should use these cp genome data to develop markers for species identification*". In S7 Table you can find information about potential candidate markers for species identification, upon the search for highly-variable regions using the software DNASP6.

3) Reviewer1 also stated that "*I thought that authors should consider to develop the InDel markers, it will be a fast procedure for species identification*". However, we do not share the idea of including InDels in the study as they are highly variable, and they mostly occur in AT-rich microsatellite sequences. For this reason, we think these sequences may not be conserved and that they are not reliable for species identification. On the contrary, polymorphisms are usually more conserved (Mader et al. 2018). For this reason, they are more and more used in genomic research. For example, recent papers like Bi et al. 2018 and Song et al. 2019 (see Reference section) carried out the search for highly-variable regions only focusing on SNPs by using a similar approach to ours.

4) Finally, reviewer1 mentioned that "*After developing, the PCR amplification should be performed for validating the InDel markers in related species.*" Although the final aim of our research is to develop primers for PCR amplification of target genetic sequences, we do not consider this feasible in 45 days, as it would take several months. We have planned to do this part of our research in the near future when a higher number of individuals per species will be tested for SNPs validation.

I am now providing you with an answer on specific comments in the paper:

- "*L109 chloroplast genes revised to chloroplast genome".* Corrected

- "*L113 in the angiosperms*". Corrected

- "*L186 check the value "160774 bp ± 602"?*". Corrected

- "*L195 Table 1 37.4±0.0, 36±0.0, 36.8±0.0".* Corrected

- "*L274 Millettia, be italic*". Corrected

- "*L455-456 How to know paraphyletic with the genus Hymenaea? No phylogenetic tree*". We realized that we do not have sufficient data to confirm what was stated by Estrella et al. 2018. Because this part of our studies does not aim to resolve the phylogeny of African trees, we removed that part from the Discussion.

- "*L491 No difference between Khaya grandifoliola and Khaya senegalensis. No indel existed between two species?*" We looked at the alignment between the two *Khaya* species (pairwise similarity=99.999%), but we only found a low number of gaps no longer than 1 bp. Therefore, no relevant InDels were found.

After your evaluation of our comments, if you think our manuscript still does not meet the PLOS ONE publication criteria, we agree with that decision and submit it to another journal. I am looking forward to hearing your opinion. Thanks for your time and consideration.

Sincerely,

Maurizio Mascarello
